# “Free Trade” for Physicians

**Published:** 1888-02

**Authors:** 


### "*FREE TRADE*" *FOR PHYSICIANS.*

We suppose that there are few physicans in the country who feel desirous of contributing to swell the surplus which the nation is puzzled to know what to do with. It is not within the province of the JOURNAL to discuss the advantages of Free Trade or Protection, but we believe we will have the sympathy of all our readers in advocating the removal of import duties on surgical and all other instruments and appliances used in the diagnosis and treatment of diseases.

Those physicians who have had the advantages of a trip to Europe, usually purchase every instrument of which they have need there, because they find them to be from twenty-five to seventy-five per cent. cheaper than ours. Surgical instruments and appliances are, in this country, so costly, that their purchase is often a heavy drain upon the physician's income; and, to many physicians in country practice, the cost of instruments makes it utterly impossible for them to supply themselves with those which, in an accident or sudden emergency, may be essential to the saving of life, or the prevention of years of suffering to the unfortunate.

We believe that the advantages to the community in allowing free competition in all supplies needed for the sick and suffering, would far outweigh any individual injury to our own manufacturers.

It will be remembered with what an outcry the proposal to place quinine on the free list was received. Manufacturers who had acquired millions, extorted from the struggling pioneers of our great West, resisted the removal of import duties, and, by specious arguments, almost persuaded the representatives of the people that they were public benefactors in supplying the nation with quinine at about four dollars an ounce. A few weeks ago, quinine was sold in New York at twenty-five cents an ounce.

The same results, though, of course, in a lesser degree, would follow the abolition of import duties on surgical instruments.

By united action on the part of the profession, it may readily be accomplished.

